# Psychometric properties of fatigue severity scale in Chinese systemic lupus erythematosus patients

**DOI:** 10.1186/s12955-019-1141-x

**Published:** 2019-04-24

**Authors:** Chenchen Feng, Qian He, Yan Wu, Xiaokun Hu, Juan Wu, Xiaoli He, Shuzhen Zhao

**Affiliations:** Outpatient Department, West China Hospital of Sichuan University, No. 37, Guoxue Lane, Chengdu, 610041 Sichuan Province People’s Republic of China

**Keywords:** Fatigue, Psychometric properties, Scale, Systemic lupus erythematosus

## Abstract

**Background:**

Fatigue is the most common symptom in Systemic Lupus Erythematosus (SLE) patients. Many fatigue instruments have been used in SLE, with Fatigue Severity Scale (FSS) mostly adopted. However, fatigue instruments haven’t been tested in the Chinese SLE population. The aim of our study was to test the psychometric properties of FSS in Chinese SLE patients.

**Methods:**

A cross-sectional study was conducted. 201 patients diagnosed with SLE were enrolled in the study with convenience sampling. Fatigue score, depression score and vitality subscale score of SF-36 were collected. Floor and ceiling effects were tested. Factor analysis was conducted. Reliability and validity of FSS were also tested.

**Results:**

Floor (4.50%) and ceiling (4.00%) effects were minimal. One factor was extracted, explaining 61.80% of total variance. When item1 and item 2 were deleted, one factor explained 69.54% of variance, and Cronbach’s Alpha increased from 0.92 to 0.93. Intraclass correlation coefficient (ICC) was 0.94. Fatigue correlated with both depression (*r* = 0.52, *P* < 0.01) and vitality (*r* = − 0.55, *P* < 0.01), indicating acceptable construct validity for original FSS. When item 1 and 2 were removed, the correlation coefficient between 7-item FSS and vitality increased (*r* = − 0.58, *P* < 0.01), while correlation coefficient between 7-item FSS and depression remained the same (*r* = 0.52, *P* < 0.01). Known-groups validity was verified by that patients with depression showed higher fatigue score both for 9-item (*Z* = -5.56, *P* < 0.001) and 7-item FSS (*Z* = -5.70, *P* < 0.001).

**Conclusions:**

9-item FSS is a reliable instrument and can be used to assess fatigue problem in Chinese SLE patients, and 7-item FSS also demonstrated good psychometric properties in the same participants.

## Background

Systemic lupus erythematosus (SLE) is a multisystem, autoimmune, inflammatory disorder presenting with symptoms from various organs, including joint, skin, kidney, brain, cardiovascular, lung, etc. [1]. In western countries and the United States, SLE prevalence was 4 to 250/100,000 in adult. In China, its prevalence was 97.5 to 100/100,000 [2–5]. The peak SLE incidence occurs at age 20–29 years, followed by 30–39 years old for females, but in 70–74 years old for males [3], and the female-to-male ratio was about 10:1 in China [6–8]. The most common symptom of SLE is fatigue, with about 51%~ 90% of SLE patients experiencing fatigue problem [9]. Fatigue can affect the daily lives of SLE patients [10, 11], and is also related to sleep disorders [12], pain, depression, and quality of life [13]. Fatigue is a subjective phenomenon without clinically validated definition [14], and it’s hard for patients with fatigue problem to participate in a long time investigation, so a reliable and simple instrument to measure fatigue problem of SLE is important, which would be conducive to interventions and outcomes monitoring.

Many fatigue instruments have been used in SLE patients. A study conducted a systematic review of the fatigue instruments used in SLE patients. 15 instruments were found, including Visual Analogue Scale [15], Fatigue Severity Scale (FSS) [16], Chalder Fatigue Scale [17], Multidimensional Fatigue Inventory (20 items) [18], etc. The study found that FSS was most widely used and recommended the use of FSS to measure fatigue for SLE patients. First developed and tested by Krupp in SLE and multiple sclerosis patients [16], FSS was used to measure the impact of fatigue on functions [9]. It is a unidimensional fatigue scale with 9 items. FSS has also been used in diseases other than SLE, such as cancer [19], chronic hepatitis C [20], central neural system disease [21], chronic neck pain [22], major depressive disorder [23], stroke [24, 25], as well as general population [26]. Though widely used in other countries, past properties test of FSS in SLE enrolled relatively small number of participants [27–29], and the FSS hasn’t been tested in Chinese SLE patients. The properties of a scale are related to specific patient group and cultural factors, so the aim of our study was to test the psychometric properties of FSS in Chinese SLE.

## Patients and methods

### Study design

A cross-sectional study was conducted in a rheumatology outpatient in one general hospital in Chengdu, China.

### Patients

Between May 2017 and July 2017, 201 patients were enrolled in the study by convenience sampling from the Rheumatic Outpatient Clinic of West China Hospital of Sichuan University. Inclusion criteria: (1) 18 to 75 years old, (2) diagnosed with American College of Rheumatology revised criteria for the classification of SLE [30], (3) having no communication problems with interviewers, (4) informed consent. Exclusion criteria: (1) with comorbid fatigue-related conditions such as cancer, obesity (BMI ≥ 28Kg/m^2^), pregnant, taking medications that affect sleep, central neural system disease, rheumatoid arthritis, etc.

### Measurements

#### Demographic and clinical variables

Demographic and clinical variables were collected from each patient, including age, gender, level of education, residence, current work status, average monthly household income, medical insurance, whether having caregiver, disease duration, disease activity assessed with Systemic Lupus Erythematosus Disease Activity Index (SLEDAI), daily glucocorticoids dose, and depression.

#### FSS

FSS was used to test patients’ fatigue severity. This instrument contains 9 items, and patients choose score from 1 (strongly disagree) to 7 (strongly agree) to indicate their agreement level to each item. The total score is the mean of 9 items’ scores, with higher score indicating higher degree of fatigue. Score 4 or higher means having fatigue [31].

#### Self-rating depression scale (SDS)

SDS was used to assess the depression of patients. It has 20 items, and all items are scored from 1 to 4 to specify the occurrence frequency. Score over 70 means severe depression, score between 60 and 69 indicating moderate to marked depressive symptoms, score between 50 and 59 meaning minimal to mild depression, and score less than 50 indicates no depression [32].

#### Sf- 36

SF- 36 was used to measure patient’s quality of life. It has 8 subscales: Physical Functioning (10 items), Role-Physical (4 items), Bodily Pain (2 items), General Health (5 items), Vitality (4 items), Social Functioning (2 items), Role-Emotional (3 items), and Mental Health (5 items). Item scores were coded, summed, and transformed. Total score ranges from 0 to 100, with higher score indicating better health status [32]. The Vitality subscale was used to test the concurrent validity of FSS.

### Data collection procedures

The study was approved by the Ethics Committees of West China Hospital of Sichuan University (2017/137). When patients visited the rheumatology outpatient clinic, rheumatologists first diagnosed SLE patients with American College of Rheumatology revised criteria. Then, rheumatologists excluded patients with comorbid fatigue-related conditions. The doctors would introduce the study to SLE patients first. When patients finished their visits, two researchers informed patients who met other inclusion criteria of the aim and procedures of the study. After giving their informed consents, patients were instructed to fill in the questionnaires. One week after their visits, two researchers made phone calls to the same participants and investigated their fatigue scores again.

### Statistical analysis

SPSS software (SPSS Inc., Chicago, IL, USA; version 17.0) was used. Floor and ceiling effects were applied to test the acceptability of the scale. Exploratory factor analysis was used to explore the dimensional structure of the FSS. Principal factor was used to extract factors, and quartimax rotation was applied. Eigen value above 1.00 and the scree plot were used to confirm the factor number. The Cronbach alpha was used to indicate FSS’s internal consistency. The test-retest reliability was determined by the intraclass correlation coefficient (ICC), which was calculated by SLE patients’ FSS scores collected at two different times. To test the concurrent validity, spearman correlation was adopted to explore the correlation between FSS score and depression score, as well as the correlation between FSS score and Vitality score. To test known-groups validity, scores difference between depressed and non-depressed patients were compared using Mann-Whitney Test. *P* < 0.05 means statistical significance.

## Results

### Demographic and clinical characteristics

A total of 201 patients participated in this study at their first visit, and 98% of them were women. The mean (SD) age was 38.5(10.9) years, and median disease duration was 6 years. The median SLEDAI score was 4. Other variables were showed in Table 1. 184 patients completed the second investigation of fatigue on the phone.

**Table 1:** Demographic and Clinical Characteristics of SLE patients

Variable	Value
Age, mean (SD)	38.5(10.9)
Gender, *n* (%)	
Female	197 (98.0)
Male	4 (2.0)
Level of education, *n* (%)	
Illiterate	15 (7.5)
Elementary and junior high school	79(39.3)
High school/vocational high school	83(41.3)
University and college	20(9.9)
Graduate school	4(2.0)
Residence, *n* (%)	
Rural area	91(45.3)
Urban area	110(54.7)
Current work status, *n* (%)	
Retired	5(2.5)
Unemployed	82 (40.8)
Employed	114(56.7)
Average monthly household income, *n* (%)	
0~2000 RMB	92(45.8)
2000~5000 RMB	89(44.3)
5000~10,000 RMB	12(6.0)
≥ 10,000 RMB	8(3.9)
Medical insurance, *n* (%)	
Yes	99(49.3)
No	102(50.7)
Whether having caregiver, *n* (%)	
Yes	169(84.1)
No	32(15.9)
Disease duration, median (range), years	6(0.08, 38)
SLADAI score, median (range)	4 (2, 45)
Daily glucocorticoids dose, *n* (%)	
≤ 7.5 mg	77(38.3)
7.5~30 mg	118(58.7)
30~100 mg	6(3)
Depression, *n* (%)	
No	112(55.7)
Mild	53(26.4)
Moderate	31(15.4)
high	5(2.5)

*SLADAI* Systemic lupus erythematosus disease activity index

### Acceptability and dimensionality

For floor and ceiling effects, 4.5% of the sample had the lowest possible score and 4% of them got the highest possible score**.** The Kaiser-Meyer-Olkin (KMO) value was 0.92, indicating the sample size was suitable for factor analysis. With the use of principal factors method and quartimax rotation, one factor was revealed, and scree plot also justified this result. One factor explained 61.80% of the total variance. Factor loadings ranged from 0.59 to 0.86 (Table 2). Item 1 and item 2 showed the lowest correlation with the rest of the items (Table 3). When we deleted item 1 and item 2, one factor was extracted, and 7 items explained 69.54% of the total variance. Factor loadings ranged from 0.67 to 0.88 (Table 2).

**Table 2:** Factor analysis for the 9-item FSS and 7-item FSS

Item	Factor loading	Factor loading
Item1(My motivation is lower when I am fatigued)	0.59	_
Item2(Exercise brings on my fatigue)	0.67	_
Item3 (I am easily fatigued)	0.70	0.67
Item4 (Fatigue interferes with my physical functioning)	0.86	0.86
Item5 (Fatigue causes frequent problems for me)	0.86	0.88
Item6 (My fatigue prevents sustained physical functioning)	0.86	0.87
Item7 (Fatigue interferes with carrying out certain duties and responsibilities)	0.85	0.86
Item8 (Fatigue is among my three most disabling symptoms)	0.85	0.86
Item9 (Fatigue interferes with my work, family or social life)	0.80	0.83
Eigenvalues	5.56	4.87
Total variance (%)	61.80	69.54
KMO	0.92	0.91

Note: *KMO* Kaiser-Meyer-Olkin value

**Table 3:** Item-total correlation for the 9-item FSS and 7-item FSS

Item	Total 9-item FSS score	Total 7-item score
Item1	0.52	_
Item 2	0.59	_
Item 3	0.62	0.58
Item 4	0.80	0.80
Item 5	0.80	0.82
Item 6	0.81	0.81
Item 7	0.78	0.80
Item 8	0.79	0.80
Item 9	0.73	0.75

### Reliability and validity

Cronbach’s Alpha showed a minor increase from 0.92 to 0.93 when item 1 and item 2 were deleted. ICC was 0.94, and there was no significant change in ICC after the deletion of items. We found significant correlation between 9-item FSS score and SDS score (*r* = 0.52, *P* < 0.01). Significant correlation between 9-item FSS score and Vitality score (*r* = − 0.55, *P* < 0.01) was also found (Table 4). The correlation coefficient between 7-item FSS score and Vitality score increased (*r* = − 0.58, *P* < 0.01), while the correlation coefficient between 7-item FSS score and depression score remained the same (Table 4). Mann-Whitney Test showed that patients with depression demonstrated higher median fatigue score both for 9-item FSS and 7-item FSS (*P* < 0.001) (Table 5).

**Table 4:** Correlation of the FSS score with depression and vitality score

Variable	FSS-9	FSS-7
Depression	0.52**	0.52**
Vitality	−0.55**	− 0.58**

Note: ** = *P* < 0.01

**Table 5:** Comparison of fatigue in patients with or without depression

	9-item FSS		7-item FSS	
	With depression (*n* = 89)	Without depression (*n* = 112)	With depression (n = 89)	Without depression (n = 112)
Median	4.80	3.35	4.43	2.57
*Z*		−5.56		− 5.70
*P* value		0.000^a^		0.000^a^

Note: SDS score ≥ 50 means depression, and SDS score < 50 means no depression

^a^Mann–Whitney test

## Discussion

FSS has been translated into Chinese version but hasn’t been applied in SLE patients. As fatigue is one of the mostly referred subjective symptoms in SLE, there is a need for a reliable instrument to assess this problem. This was the first study to test the psychometric properties of FSS in Chinese SLE patients. Our study showed that original 9-item FSS had acceptable psychometric properties, in accordance with findings from other language versions in different populations [28, 33, 34]. And the 7-item FSS also have good psychometric properties for Chinese SLE patients.

When more than 15% of the samples have either the lowest or the highest score, the instrument demonstrates floor and ceiling effects [35]. In our study, 4.5% of the samples had the lowest score and 4% had the highest score, indicating the ceiling and floor effects were minimal. However, Ferentinos’ study [36] demonstrated a ceiling effect of FSS in major depression subjects. Possible explanation may be that depressed patients are more sensitive to negative feelings and experiences so they are more likely to choose higher score of FSS. Such result indicated that the acceptability of the FSS differs in different patient group, which further confirmed the importance to test the psychometric properties of an instrument before applying it to a specific disease.

Exploratory factor analysis results were in agreement with previous studies using FSS in SLE patients [27–29]. Only one factor was extracted in our sample, explaining 61.80% of the total variance. All the item loadings were higher than 0.40. The first and second item showed the lowest correlation with the rest of the items, in line with Lerdal’s report [26]. Ottonello’s study [37] reported that deletion of item 1 could improve the unidimensionality of the FSS in Italian MS patients. Lerdal’s study concluded that the removal of item 1 and item 2 could improve FSS’s psychometric properties in MS and stroke patients [14, 38], while Mill’s study [39] concluded that a 5-item FSS scale could measure the impact of the fatigue better than the original 9-item scale in MS patients. Wang’s study indicated that the deletion of item 2 and item 3 showed better psychometric properties in major depressive disorder participants [23]. When we deleted item 1 and item 2, 7 items explained 69.54% of the total variance.

Past studies demonstrated that the Cronbach’s Alpha of FSS ranged from 0.87 to 0.96 [34, 40–43] in different language versions and different disease subgroups. The original FSS had good internal consistency. Our study found that the Cronbach’s Alpha showed a minor increase from 0.92 to 0.93 after the deletion of item 1 and item 2. Possible reason may be that the description of item 1 and item 2 can’t measure the severity of fatigue, and they lack the discrimination ability for healthy and disease-suffered groups since healthy people can also have such experiences. The test-retest reliability was good, indicating that it was a stable instrument to use at different interval.

The original FSS showed acceptable construct validity. Significant correlation between FSS score and depression score (*r* = 0.52, *P* < 0.01) was found, in line with past studies showing that the correlation coefficient ranged from 0.46 to 0.75 [40, 44, 45]. What’s more, correlation was also found between FSS score and score of Vitality (*r* = − 0.55, *P* < 0.01), in accordance with previous reports, in which the correlation coefficient ranged from − 0.32 to − 0.72 [27, 34, 46]. After the deletion of the item 1 and 2, the concurrent validity seemed better since the correlation coefficient between 7-item FSS score and Vitality score increased (*r* = − 0.58, *P* < 0.01), while the correlation coefficient between 7-item FSS score and depression score remained the same (*r* = 0.52, *P* < 0.01) (Table 4).

Known-groups validity was tested by comparing fatigue difference between patients with and without depression. We hypothesized that patients with depression would show higher degree of fatigue. And our result verified such hypothesis, showing FSS’s suitable ability to reflect differences between two groups both for original FSS and 7-item FSS (Table 5).

Our study did have some limitations. Firstly, we only investigated SLE patients in one general hospital with a small number of male patients, and the female to male ratio was less than the reported ratio in Chinese SLE patients, so the representativeness of the samples may be reduced. Secondly, the overall disease activity of SLE was mild, so further investigation of SLE with different level of disease activity are necessary to increase study’s generalizability. Finally, depression can have some impacts on the fatigue problem of SLE patients. Our study didn’t exclude the depressive patients as depression and fatigue can be interactive in SLE patients and it may be impractical to distinguish these two symptoms in SLE patients.

## Conclusion

In summary, 9-item FSS is a reliable instrument and can be used to assess fatigue problem in Chinese SLE. The 7-item FSS also demonstrated good psychometric properties. As the deletion of two items simplify the content of FSS, it may make patients more willing to participate in study. However, another study about whether the 7-item FSS is more easy to use from patients’ perspectives is needed.

## Abbreviations

FSS: Fatigue severity scale
ICC: Intraclass correlation coefficient
SD: Standard deviation
SDS: Self-Rating depression scale
SLE: Systemic lupus erythematosus
SLEDAI: Systemic lupus erythematosus disease activity index
